# Supplementary material for: Synthesis and Physico-Chemical Analysis of Dextran from Maltodextrin via pH Controlled Fermentation by Gluconobacter oxydans
Source: Foods. 2025 Jan 1;14(1):85. doi: 10.3390/foods14010085 (PMC11719824; doi:10.3390/foods14010085)
Supplement: Supplementary file 1 [file foods-14-00085-s001.zip › foods-3342768-supplementary.pdf]

Synthesis and Physico-Chemical Analysis of Dextran from Maltodextrin via pH Controlled Fermentation by *Gluconobacter oxydans*

Seung-Min Baek [1,†], Bo-Ram Park [1,*,†], Legesse Shiferaw Chewaka [1], Yun-Sang So [2], Ji-Hye Jung [1], Seul Lee [1] and Ji Young Park [1]

[1] Fermentation Research Department, National Institute of Agricultural Science, RDA, Jeonju 54875, Republic of Korea
[2] Department of Food Science & Biotechnology and Carbohydrate Bioproduct Research Center, Sejong
University, Seoul 05006, Republic of Korea
* Correspondence: bboram27@korea.kr
† These authors contributed equally to this work.

Supplementary file

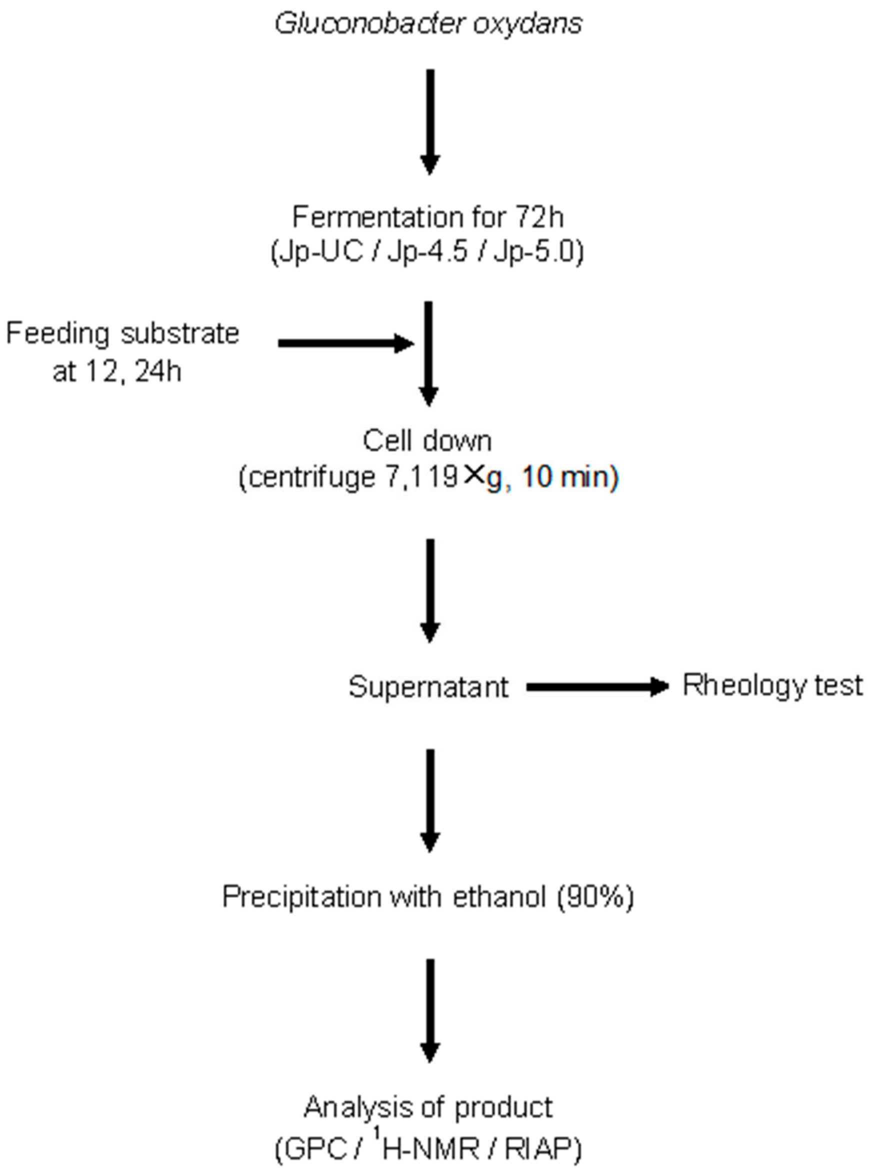

Figure S1. Schematic diagram of Dextran Production Process by *Gluconobacter oxydans*
